# Early childhood precursors for eating problems in adolescence: a 15-year longitudinal community study

**DOI:** 10.1186/2050-2974-1-35

**Published:** 2013-08-20

**Authors:** Gertrud Sofie Hafstad, Tilmann von Soest, Leila Torgersen

**Affiliations:** 1 Division of Mental Health, Norwegian Institute of Public Health, P.O. Box 4404, Nydalen, 0403, Oslo, Norway; 2 NOVA, Norwegian Social Research, P.O. Box 3223, Elisenberg, 0208, Oslo, Norway; 3 Department of Psychology, University of Oslo, P.O. Box 1094, Blindern, 0317, Oslo, Norway

**Keywords:** Prospective study, Longitudinal, Eating problems, Disordered eating, Sleep problems, Early childhood

## Abstract

**Background:**

This longitudinal community study investigated the role of individual risk factors in early childhood (before age five) for the development of eating problems in adolescence. Nine hundred twenty-one mothers completed the first questionnaire when their child was 1.5 years old, and again when their child was 2.5 (*n* = 784) and 4.5 (*n* = 737) years old. Three hundred seventy-three of these children completed the Eating Attitudes Test (EAT) when they were 16 years old.

**Results:**

Mother-rated early childhood sleep problems (assessed before the age of five) predicted self-rated eating problems in adolescents, with gender, birth weight, and a number of early childhood internal and environmental factors controlled. Unexpectedly, early childhood eating problems were not associated with later eating problems.

**Conclusions:**

The possible role of sleep in the development of eating problems needs further investigation. In particular, mediating mechanisms should be studied more closely.

## Background

Eating problems normally onset in adolescence, with a peak at about 15-16 years [1]. Although a number of risk factors for eating disorders and eating problems have been identified [2-4], the earlier childhood precursors of adolescent eating problems are not well understood. Existing longitudinal studies on risk factors for eating problems have generally relied on relatively short time spans [5] and have reported that the best predictor of late adolescence eating problems was an already present eating problem score at age 12 [5]. This shows the need for studies starting at an earlier age to obtain information about potential risk factors at an age where such eating problems are not yet firmly established. The aim of this study was therefore to identify early childhood predictors of eating problems in adolescence at age 16.

Problematic eating patterns in childhood may develop to more serious eating problems during adolescence and may therefore be of particular importance. However, so far, only a small number of prospective studies have assessed eating behavior in younger children (under the age of 10 years) and followed the respondents into adolescence. In one of these studies, Kotler, Cohen, Davies and collaborators [6] showed that negative affect at mealtimes, struggles with food, and eating conflicts in early childhood predicted eating problems in adolescence, whereas five other types of problematic eating behaviors – including picky eating – were not predictive. Moreover, no longitudinal association was reported between childhood picky eating and eating concerns at age 14 in another study conducted by Martin and collaborators [7]. In yet another prospective study, however, it was shown that picky eating in young children was related to adolescent eating problems [8]. Thus, even though some prospective studies indicate certain eating problems in early childhood to be related to adolescent eating problems, the studies do not provide firm conclusions concerning which problems are predictive. Particularly for picky eating, some studies show long-term associations with adolescence eating problems, whereas other do not.

There is an ongoing discussion whether adolescent eating problems best can be understood as eating concerns and dieting escalating into more serious eating problems, or whether eating problems rather is a way of handling earlier manifestations of emotional problems. According to the affect regulating model [9] people may binge eat in an effort to provide comfort and distraction from adverse emotions. Moreover, research has shown that negative affect has been found to be prospectively related to eating problems among 9-10 year olds [10,11] as well as in adolescents [5,12].

Starting the examination of risk factors in toddlerhood may entail some challenges concerning assessment of child problems, and child behavioral descriptions will often be limited to parent report. As such, early manifestations of negative affect may be observed by the mothers of the toddlers as difficult temperament. Childhood temperament in toddlerhood has previously been described an early sign of later negative affect or emotional problems [13]. Relevant for the current study, one longitudinal study investigated the role of child temperament in the development of eating concerns in early adolescence [7] and found that a high level of negative affectivity in 3–4-year-old girls was associated with adolescent eating concerns. Moreover, difficult infant temperament has been concurrently associated with negative mealtimes and food refusal in young children [14]. Feeding difficulties have also been found to be more prevalent in children who are described as less sociable, difficult or demanding, and parental reports of having a shy, emotional child have been related to children’s unwillingness to try new foods [14,15]. Several longitudinal studies in adolescence [5-16] have identified negative emotionality and depressive mood as predictors of eating problems, suggesting that it would be useful to extend the search downwards in age in order to possibly identify at which age emotionality, other temperamental traits, and internalizing problems may first be identified as risk factors for eating problems.

Finally, it has been suggested that early eating problems may be part of a self-regulation cluster, encompassing, for instance, difficulties with sleep and emotion regulation, which again may serve as precursors of a number of different emotional problems [17]. Childhood sleep problems are closely related to eating problems in early childhood [18]. Thus, it is reasonable to believe that sleep problems early in life could be a precursor, or at least a correlate to later eating problems. We were therefore interested in studying the potential longitudinal link between sleep and eating concerns.

Overall, there is limited research on childhood predictors of adolescent eating problems, as research on early childhood risk factors has mainly relied on retrospective information from cross-sectional studies or on designs measuring outcomes before adolescent eating problems occur. In the present study we seek to build on the previous body of work by extending the investigation of possible precursors downward to early childhood, i.e., before school age. More specifically, we aimed to investigate prospective predictors of adolescent eating problems, including eating problems, temperament, internalizing problems, and sleep problems in preschool age. Based on existing research, we hypothesized that picky eating, sleep problems, temperamental shyness and emotionality assessed before 5 years would predict adolescent eating problems.

## Methods

### Participants and procedures

The sample consisted of Norwegian children and their mothers participating in a longitudinal community survey study (The Tracking Opportunities and Problems – TOPP study) conducted in Norway between 1993 and 2009. The study was designed to examine a broad array of somatic and mental health variables. All families from 19 geographical health care areas in eastern Norway who brought their child to a health clinic in 1993 for their 18-month-old checkup were invited to complete a questionnaire. In Norway, more than 95% of families with children visit a child health clinic eight to 12 times during the child’s first four years of life. Of 1,081 eligible families, 913 mothers (84%) completed the questionnaire as the child was 18 months old. The families were followed through subsequent health clinic visits when the children were 2.5 years old and 4.5 years old. At the second data collection (2.5 years), 777 mothers (85% of those participating at baseline) participated, whereas 727 (80%) participated as the child was 4.5 years old. Beyond this, postal follow-ups of the mothers were conducted when the children were 8, 12, 14–15, and 16 years old. From the age of 12 on, the children’s self-reports were also entered into the study, along with their mothers’ continuing reports. In the present study, we used mothers’ reports from the first three data collection waves (i.e., child aged 1.5, 2.5, and 4.5 years), and child reports from the last wave (adolescents aged 16 years, *N =* 373). All participating mothers provided written consent before entering the study. Mothers also provided written consent for their adolescent children to participate in the study from age 12 onwards. The study was approved by the The Regional Committee for Medical and Health Research Ethics. This was an ethnically homogeneous sample, with more than 95% of the families being ethnic Norwegians. Of those recruited at baseline, 44% remained in the study at the last measurement point (age 16). Background data from the child health clinics showed that non-respondent mothers at baseline did not differ significantly from responding mothers in age, education, employment status, or marital status [19]. Attrition analyses from baseline to the last measurement point showed that low maternal educational level, but not other variables (such as mother's temperament and psychological distress, child's temperament, and mothers' emotional support from partner and friends) predicted drop-out [20]. Moreover, boys were less likely to participate in the final wave than were girls, and the attrition from the first wave was disproportionately high among boys. Associations between variables at baseline did not differ among families who dropped out those who remained in the study, suggesting that estimated associations between variables are generalizable [20].

### Measures

All early childhood measures were pooled across the three measurement points, i.e. at child age 1.5, 2.5, and 4.5. The pooling procedure was conducted for two purposes: 1) to ensure the stability of the phenomenon measured over the preschool years. Such behaviours normally fluctuate at this age, and by pooling the measures we attempted to avoid that unsystematic fluctuation would influence the analyses, and 2) to increase the internal reliability of the measure, as the pooling procedure increased the number of items used to assess each phenomenon.

### Parent-completed measures

#### Picky eating in childhood

At the child’s ages 1.5, 2.5, and 4.5 years, mothers completed the Behavior Checklist (BCL) [21]. The BCL consists of 19 items covering 12 behavioral categories. The two eating-related items in this scale, assessing lack of appetite and food fussiness, were used as a measure of picky eating. The items have three response options ranging from 1 (Usually has a good appetite/Not picky about eating) to 3 (Nearly always has a poor appetite/Very picky, doesn’t like many different foods). The two items were collapsed over all three time points (1.5 to 4.5) into one mean score indicating eating problems before age five. Higher mean scores indicated greater degree of problematic eating. Cronbach’s α of the pooled score was .70.

#### Sleep pattern in childhood

Three items from the BCL were used at the first three time points: difficulties falling asleep at bedtime, nightly awakenings, and unwillingness to sleep alone. Again, the items had three response options (e.g. “Hardly sleeps at all” to “Sleeps much of the time” and “Very rarely wakes up at night” to “Often wakes up at night”) and all three items were collapsed over all tree time points into one mean score, with higher values indicating greater difficulties with sleep before age five. The measure had an acceptable internal consistency, with a Cronbach’s α of .74.

#### Internalizing problems in childhood

Internalizing problems were measured by three items from the BCL covering the two domains worried and fearful. Again, the three items were collapsed over all three time points, and higher scores indicated a greater degree of internalizing problems. Cronbach’s α for this scale was .71. Results from previous research with this scale have suggested that it is a valid assessment of internalizing problems [22,23].

Childhood temperament was assessed at the first three time points with two subscales from the 20-item Emotionality, Activity, Shyness and Sociability Temperament survey for children (EAS) [24]. The present study utilized two temperamental dimensions: shyness (e.g., Child takes a long time to warm up to strangers) and emotionality (e.g., Child gets upset easily), both covered by five items. Parents responded on 5-point Likert scales indicating how characteristic the behaviors were of their child. Mean scores were calculated for each subscale over all three time points, with possible subscale scores ranging from one to five. This measure has been found to have adequate psychometric properties for ages 1.5 to 4.5 years [18]. Cronbach’s α for the pooled scales was .86 for shyness and .82 for emotionality.

### Outcome measure

#### Self-reported eating problems in adolescence

At age 16, a 12-item version of the Eating Attitudes Test (EAT-12) was used to measure problems and concerns related to dieting, bulimia and food preoccupation, and oral control [25,26]. The EAT-12 has been used in several other Norwegian adolescent samples, and the psychometric properties in Norwegian samples have been well established [5,16,26,27]. Participants responded on scales ranging from 0 (Never) to 3 (Always). A mean score for the 12 items was calculated, with higher scores indicating more serious eating problems. The internal consistency of the scale in this sample was α = .82.

### Covariates

#### Gender

The child’s gender was reported by mothers at all measurements points and coded boys = 0 and girls = 1.

#### Birth weight and perinatal complications

The child’s birth weight was reported by the mothers at child age 1.5. They also indicated (yes or no) whether they had any perinatal complications (“Did you experience complications during delivery?”).

#### Physical health in childhood

Physical health before the age of five was measured at the three first time points by an item that assessed whether the child had chronic physical impairments, such as a hearing impairment, cerebral palsy, heart disease or cleft lip/palate. The items had yes and no response options, and whose parent provided a “yes” response at any of the three time points were considered as having a physical impairment before age 5. The stability of the responses was .59 between age 1.5 and 2.5 and .39 between age 2.5 and 4.5.

### Statistical analyses

Means and standard deviations were calculated for adolescent eating problems and for the preschool age predictors where appropriate. We computed bivariate Pearson correlations for all variables to determine concurrent childhood associations as well as the extent to which early childhood variables were related to later eating problems. Missing values were replaced by the scale mean. To examine gender differences in eating problems, we conducted independent sample *t* tests. We conducted multiple regression analyses with simultaneous entry of variables to investigate whether there was a unique association of any childhood variable on adolescent eating problems when controlling for all other predictors. Due to the potential non-normal distribution of the EAT scores, a bootstrapping procedure was used to compute standard errors of the estimates in all regression analyses. We conducted additional analyses to examine gender differences in the association between predictors and eating problems. For this purpose, we performed one regression analysis for each predictor, where gender, the predictor, and the interaction term between gender and the predictor were simultaneously included as independent variables to predict adolescent eating problems. Because several physical conditions, as well as childhood weight status may influence eating behaviors [28,29] we controlled for physical illness, perinatal complications, and birth weight in the analyses.

## Results

Predictor variables used in the current study were all assessed before age five, and thus before the adolescent eating problems were assessed. Table 1 presents descriptive statistics. Overall, children were reported to have a mild to moderate degree of eating problems over the preschool years, being rated as sometimes being picky and having a reduced appetite, on average. The same was true for sleep problems, which had a similar mean level as eating problems. Temperament scores indicated that mothers on average did not perceive extremely shy temperament to be typical for their child, and that they perceived emotional temperament to be quite typical for their child. The EAT-12 scores were relatively low in this sample, with an average score of 0.57 (*sd =* .46) indicating that across the sample, most adolescents reported these problems seldom or hardly ever. As would be expected in such a community sample, the distribution of the scores was positively skewed, with most of the scores on the lower ranges.

**Table 1:** Summary statistics for all variables included in the study

	**N (%)**	**M**	**SD**	**Possible range**	**Percentiles**		
					25.	50.	75.
**Outcome measure**							
Eating problems age 16		.57	.45	0-3	.25	.50	.83
**Early predictors (age 1.5-4.5)**							
Picky eating		1.08	.46	0-2	.083	1.00	1.33
Sleep problems		1.02	.38	0-2	.077	1.00	1.22
Internalizing problems		.90	.34	0-2	.066	.89	1.11
Shyness		2.10	.55	1-5	1.70	2.06	2.40
Emotionality		2.81	.52	1-5	2.86	3.20	3.53
**Covariates**							
Gender (girls = 1)	220 (58.7)						
Birth weight (in gram)		3.550	610		3330	3570	3940
Perinatal complications (yes)		172 (18.7)					

Only 1.3% of the sample had a mean EAT score of equal or larger than 2 (reporting the eating problems as occurring often or always). This indicates that the eating problems reported in this sample were by and large mild, and may be seen as a variation of more or less serious normal eating behavior. The EAT-12 scores differed significantly by gender, with girls reporting higher levels of eating problems than boys (*t =* 7.22, *df =* 340, *p < .*001).

### Associations among key study variables

Bivariate correlations among all variables included in the study are presented in Table 2. Due to the preponderance of girls who endorse eating-problem symptoms, we first ran a set of interaction analysis to assess whether correlations with eating problems would differ significantly by gender. No significant interactions were detected, and thus we decided to conduct all analyses for boys and girls collectively. As the table shows, childhood picky eating was significantly associated with a number of other concurrently assessed variables. Mothers who reported more pickiness in their children, were more likely to report sleep problems, internalizing problems, and health problems, and somewhat less likely to report temperamental emotionality and shyness. Mother-rated child emotionality was the temperamental dimension that related to most difficulties at this early age, including sleep problems, eating problems, internalizing problems, and shyness. As for the longitudinal associations, sleep problems was the only early childhood factor that was significantly related to eating problems at age 16.

**Table 2:** Bivariate correlations among all study variables

	**1**	**2**	**3**	**4**	**5**	**6**	**7**	**8**	**9**
1. Eating problems age 16	–								
2. Picky eating	.00	–							
3. Sleep problems	.11**	.41**	–						
4. Internalizing problems	.00	.48**	.45**	_					
5. Shyness	-.02	.18**	.10**	.02	_				
6. Emotionality	.00	.15**	.14**	.23**	.17**	_			
7. Gender	.35**	.06	.09*	.06	.09*	.06	_		
8. Birth weight	.04	-.11**	-.04	.18**	.04	-.08*	-.08*	–	
9. Perinatal complications	.01	-.03	-.05	-.09*	.02	-.05	.00	.05	_
10. Physical impairments	.04	.06	-.05	.08*	.06	-.05	.03	-.11	.12*

Note. *p < 05, **p < .01, *n* age 1.5-4.5 = 727, *n* age 16 = 373, boys = 0, girls = 1.

### Multivariate analyses of childhood factors predicting eating problems in adolescence

We conducted multiple regression analyses to examine whether early childhood variables predicted eating problems in adolescence (Table 3). Birth weight, perinatal complications and functional impairment were not associated with the key variables in the study, and were therefore not kept as control variables in the multiple regression analyses. As presented in Table 3, children rated high on sleep problems before age five were significantly more likely to score high on self-reported eating problems at age 16. None of the other early childhood factors assessed predicted later eating problems in this analysis. Because of the gender differences in the level of EAT-12, we conducted additional analyses to test whether the association between sleeping patterns and eating problems was moderated by gender. No such interaction effect was identified, *β* = .07, *SE =* .13, *p >* .05. Likewise, we tested for gender differences in the relationship between all the other independent variables and eating problems. Again, there were no significant differences (all *p >* .05). Collinearity statistics revealed no sign of multicollinearity that could seem to obscure the findings (Variance Inflation Factors (VIF) for all predictor variables 1.11 or lower).

**Table 3:** Multiple regression analyses with eating problems at age 16 as the outcome variable

	**B**	**SE**	**β**	**p**
Picky eating	–.037	.067	–.031	.580
Sleep problems	.143	.077	.102	.049
Internalizing problems	-.010	.097	-.006	.914
Shyness	–.077	.042	–.097	.068
Emotionality	.009	.048	.010	.852
Gender	.324	.047	.348	.000

Note: R^2^ = .13. Gender is coded as follows: boys = 0, girls = 1.

## Discussion

In this longitudinal community study, we examined potential childhood precursors of eating problems in adolescence. The findings suggest that problematic sleep patterns before the age of five predict adolescent eating problems at age 16. However, against prediction, picky eating during early childhood did not predict later eating problems in this study. The same was true for temperament, which has previously shown to be associated with the development of eating problems. This is one of very few studies to examine the association of early childhood factors other than eating or feeding difficulties on eating problems later in life for an exception, see Martin and collaborators [7]. To the best of our knowledge, this is the first study to show a longitudinal link between sleep problems in early childhood and eating problems in adolescence.

A problematic sleep pattern in toddlerhood was not only associated with concurrent picky eating, but most notably also predicted adolescent self-reported eating problems 15 years later. The concurrent findings correspond to a growing literature on childhood eating and feeding problems suggesting that such problems should not be considered in isolation but rather as part of a cluster of self-regulation problems [14,18]. Furthermore, a high symptom load on this cluster may constitute vulnerability to certain emotional problems later in life [17]. There are indications from clinical studies that infant sleep problems may persist into the preschool and school-aged years, and that untreated sleep problems in children may have widespread negative effects on their health and functioning. For example, there is some evidence of elevated rates of behavioral problems in children with sleep problems [30]. However, the mechanisms underlying the associations between childhood eating problems and other emotional and behavioral problems are still unclear, and future research should aim to elucidate these mechanisms. Meanwhile, the current finding lends some support to the notion that psychological factors that are not strictly eating related may also give rise to disordered eating. Somewhat surprisingly, we did not find picky eating in early childhood to be predictive of later eating problems. Thus, we did not find support for the idea that eating-related problems in early childhood gradually develop into more severe eating problems in adolescence. Studies specifically examining picky eating have found this behavior to be relatively stable across childhood [31], and also to be predictive of symptoms of anorexia and bulimia in adolescence [32]. Nevertheless, our finding corresponds with research by Martin et al. [7] and Kotler et al. [6], who also failed to find an association between picky eating and later eating problems in two prospective studies of population-based samples of boys and girls. It is plausible that the pathways through which picky eating influences later eating problems are indirect, possibly mediated by dieting or other forms of dietary restraint. This may explain why we did not see a direct association over the substantial time span examined in this study. Moreover, much of the previous research has relied on samples exhibiting clinically significant eating problems in early childhood [17] or adolescence [6]. Hence, it is possible that the developmental pathways for eating problems differ for normal and clinical populations.

In accordance with previous findings, children rated high on temperamental shyness and emotionality in our study also reported having more problematic eating behavior in childhood [15]. However, this relationship did not seem to persist in our sample, as temperament was not associated with later eating problems in adolescence. The finding contrasts with findings of Martin and colleagues [7], i.e. that a difficult temperament at age four predicted eating problems ten years later. Importantly, that study analyzed extreme types of eating problems, whereas we examined the whole range of eating problems in adolescence. It is possible that adolescents with extreme eating problems have more (or more visible) problems earlier on, and thus they are easier to identify. The current study also examined a slightly longer time span, which may contribute to explain some of the discrepancies in findings. To sum up, we need more prospective studies covering the long time span from early childhood into adolescence to further disentangle the relationship between negative affectivity and eating problems.

In accordance with most studies in the field, we found that adolescent girls reported more eating problems compared to boys. However, we found no gender differences in strength of associations between predictor variables and disordered eating and data were, therefore, analyzed for both genders combined. The missing moderation effect of gender corresponds with other research indicating that factors associated with disordered eating among boys are similar to those found with girls [33].

Some limitations of this study warrant consideration. The data used in this study provide no information about diagnoses of eating disorders, making it difficult to conclude whether the results are valid only for eating problems in the normal range, or whether they can be applied to diagnosed eating disordered patients as well. Another limitation is that BMI was not assessed in this study. As picky eating may be related to smaller body size in children who later become thinner adults, this may explain the lack of relationship between picky eating and later EAT scores. Some of the predictors used in the study are comprised of few items, such that the internal consistency at each time point for these measures was rather low. To increase reliability and to obtain measures with relatively high stability through childhood, measures of the same construct from the first three time points were merged to one variable. Even though this strategy ensured acceptable reliability of the predictors, it would have been preferable to assess predictors with more comprehensive scales at each time point. We did not examine potential environmental, family and parental predisposing factors for either early childhood or more long-term problem behaviors. Factors such as family functioning and parenting from infancy forward, and peer relations from childhood forward, could be influential. Examining possible mediating factors between ages 4.5 and 16 could have contributed to explain the longitudinal relationships identified in the study. However, this paper only sought to identify early child characteristics, and future research should more closely examine the mediating mechanisms by which early self-regulation may influence the development of eating problems. Finally, the longitudinal effect sizes in this study are rather small (β = .11 in multiple regression analyses for sleep problems), and the significant predictor only accounted for a small amount of the variance in adolescent eating problems. Given the time span between the childhood assessments and the adolescent assessment, as well as the fact that our childhood behavioral data relied on mothers’ reports but the adolescent data came directly from the adolescents, even modest associations are interesting and potentially important. Moreover, explaining even a small percentage of the variance in eating problems is important, especially in a nonclinical population where base rates of eating problems are relatively low. It is important to highlight that the etiology of eating problems is multifaceted, and thus it could not be expected that one predictor would explain a large amount of the variance.

## Conclusions

Overall, the findings suggest that an increased awareness of the potential for developing eating problems can be gained by more closely monitoring children with problematic sleep patterns early in life. Given the novelty of these findings, replication is needed, and future research should also address mediating factors that can help explain the mechanisms by which these associations evolve.

## Competing interests

The authors declare that they have no competing interests.

## Authors’ contributions

TvS, LT, GH designed the study, GH conducted the statistical analysis, and drafted the manuscript. All authors read and approved the final manuscript.
